# The first case of lipoprotein glomerulopathy complicated with collagen type III glomerulopathy and literature review

**DOI:** 10.1007/s40620-022-01491-x

**Published:** 2022-11-12

**Authors:** Huixia Liu, Changqing Luo, Zhenqiong Li, Chun Zhang, Jing Xiong

**Affiliations:** Department of Nephrology, Union Hospital, Tongji Medical College, Huazhong University of Science and Technology, 1277 Jiefang Avenue, Wuhan, 430022 Hubei China

**Keywords:** Lipoprotein glomerulopathy, Collagen type III nephropathy, COL4A, Hereditary nephropathy comorbidities

## Abstract

Lipoprotein glomerulopathy (LPG) is a rare autosomal dominant kidney disease caused by pathogenic mutations in the *APOE* gene. Collagen type III glomerulopathy (CG) is a sporadic condition in adults characterized by abnormal accumulation of type III collagen in the subendothelial space and mesangium of the glomerulus. We report the first case of both LPG and CG in a 21-year-old male. A search of the literature found no confirmed reports of these two concomitant nephropathies. The patient presented with hypertension, proteinuria, hematuria and hyperlipidemia. Renal pathology showed lipid vacuoles in the enlarged glomerular capillary loops and type III collagen in the segmental mesangial area and on the inner side of the glomerular basement membrane by electron microscopy. Whole-exome sequencing revealed a heterozygous mutation (c.127C>T; p. Arg43Cys) in exon 3 of the *APOE* gene, known as the APOE-Kyoto of LPG. In addition, two heterozygous COL4A4 mutations (c.4715C>T in exon 47 and c.5065 T>C in exon 48) were observed, the first one was suspected pathogenic and the other one was uncertain significant. There is no special treatment for these diseases. The patient was treated with lipid-lowering agents, renin–angiotensin–aldosterone system inhibition and tripterygium glycosides. The patient received double-filtration plasmapheresis and immunoadsorption therapy when renal function deteriorated dramatically. Immunoadsorption was beneficial for this patient.

## Introduction

Lipoprotein glomerulopathy (LPG) is an autosomal dominant disease characterized by the histopathological feature of lipoprotein thrombi in the inner layer of glomerular capillaries and elevated serum apolipoprotein E (Apo E) levels. LPG is caused by mutations in the *APOE* gene that disturb lipoprotein metabolism. More than fifteen *APOE* gene mutations and approximately 200 cases have been reported [1]. The *APOE* gene is located on chromosome 19q13.2 and serves as a ligand for the low-density lipoprotein (LDL) and cell surface receptors of the LDL receptor gene family. LPG is mainly found in adults, especially males.

Collagen type III glomerulopathy (CG) is a rare, non-immune-mediated glomerular disease characterized by abnormal deposits of type III collagen in the glomerular mesangium and subendothelial regions and elevated serum N-terminal propeptide of type III pro-collagen (PIIINP) levels. Renal biopsy is the only method to diagnose CG. CG may have a genetic basis, but in many cases there is no evidence of familial or genetic correlation and it is considered a sporadic disorder. Approximately 100 cases have been reported [2]. The disease has no gender preference and involves all age groups from children to adults [3].

In this study, we report the first case of association between the two diseases (LPG & CG). No reported cases of the simultaneous presence of these two nephropathies can be found in the literature.

## Case report

A 21-year-old Chinese male was admitted to our hospital with hypertension in 2015. Laboratory examinations revealed nephrotic range proteinuria, hematuria, hypoproteinemia, and hyperlipidemia. Serum creatinine was in the normal range. Common secondary kidney diseases were excluded; family history was negative.

**Fig. 1:**
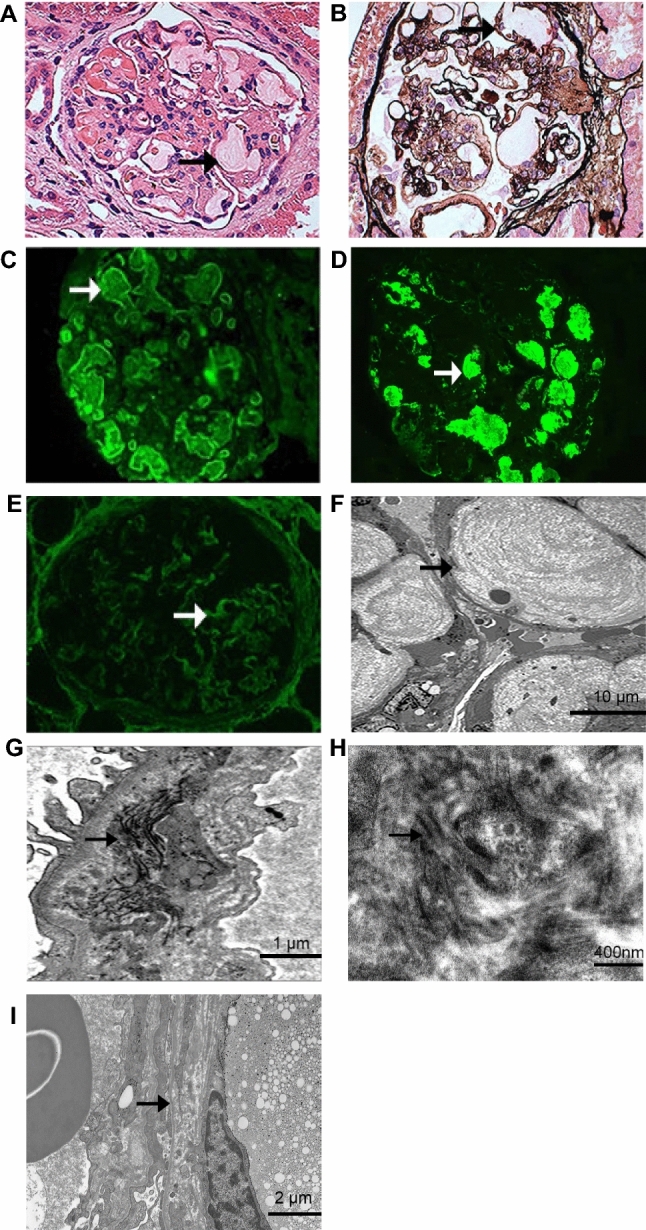
The renal pathology images and the dynamic changes in various parameters of this patient. Light microscopy images (**A**, **B**). **A** Paraffin sections with PAS staining showed that capillaries were distended by pale lipoprotein thrombi (black arrow) with positively stained material (× 400). **B** PASM+HE staining showed diffuse basement membrane stratification (black arrow) with mesangial insertion (× 600). Immunofluorescence images (**C–E**). **C** Immunofluorescence showed positive Apo B staining (white arrow) in glomerular capillaries (× 400). **D** Immunofluorescence showed positive Apo E staining (white arrow) in glomerular capillaries (× 400). **E** Collagen III (white arrow) was deposited segmentally along the glomerular basement membrane and mesangial area (× 400). Electron microscopy images (**F**–**I**). **F** Electron microscopy showed expansion of the glomerular capillary by lipoprotein thrombi (black arrow) (scale bar, 10 μm). **G**, **H** Collagen fibrils (black arrow) deposited in endothelial and mesangial regions with foot process fusion (1 μm; 400 nm). **I** Diffuse basement membrane stratification (black arrow) with mesangial membrane insertion (1 μm)

A renal biopsy was performed. Under light microscopy (LM), the capillaries were distended by pale lipoprotein thrombi that had a vague laminated appearance. The thrombi were weakly PAS-positive (Fig. 1A). Diffuse basement membrane stratification with mesangial insertion was observed (Fig. 1B). Immunofluorescence showed positive staining for APOB and APOE in the glomerular capillaries (Fig. 1C, D). Collagen III was deposited segmentally along the glomerular basement membrane (GBM) and mesangial area (Fig. 1E). The GBM was positive for α3 and α5 staining without splitting and deletion. Under electron microscopy (EM), the glomerular capillary lumens were occupied with lipoprotein thrombi (Fig. 1F). Type III collagen fibers could be seen in the segmental mesangial area and on the inner side of the GBM. The diameter of the fibers was between 60 and 100 nm, and some had periodic striations (Fig. 1G, H). The foot processes of podocytes were widely fused. Extensive mesangial insertion resulted in layered changes in the GBM. The thickness of the GBM was uneven, and delamination and tearing could be seen in some segments (Fig. 1I). The biopsy showed that the patient had LPG combined with CG. We also detected elevated serum PIIINP (90.8 ng/ml) and Apo E (8.79 mg/dL) levels.

**Fig. 2:**
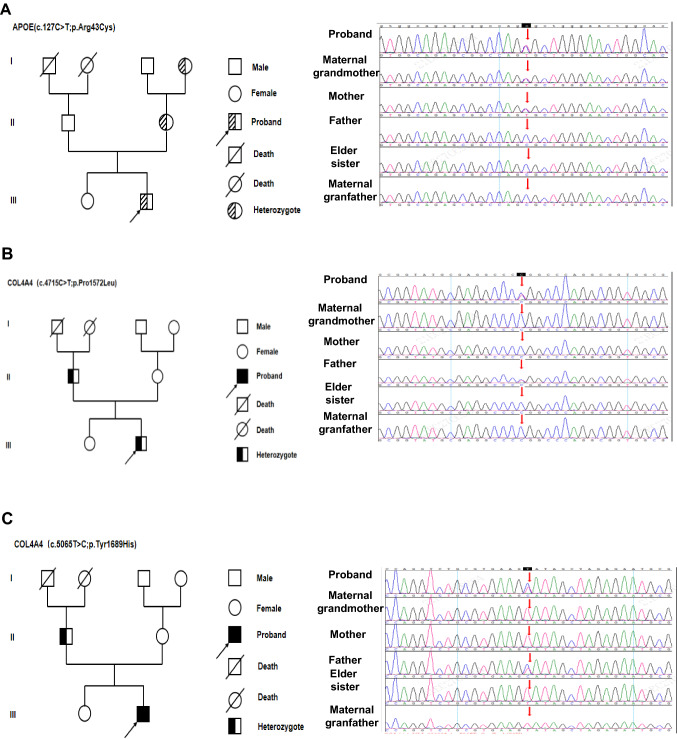
Pedigree of the family and Sanger sequencing validation. **A** Family pedigree 1; the c.127C>T; p. Arg43Cys variant was inherited from his maternal grandmother and mother by sequencing validation. **B** Family pedigree 2; the c.4715C>T; p. Pro1572Leu variant was inherited from his father. **C** Family pedigree 3; the c.5065T>C; p.Tyr1689His variant was inherited from his father

To further identify mutant genes, whole-exome sequencing (WES) was carried out for the patient, and Sanger sequencing was performed for the family members (Fig. 2A–C). Sequencing detection revealed a heterozygous mutation (c.127C>T; p. Arg43Cys) in exon 3 of the *APOE* gene, known as the APOE -Kyoto of LPG [1, 4–6]. It is a pathogenic mutation and was inherited from his grandmother and mother. Unexpectedly, two heterozygous *COL4A4* mutations (c.4715C>T in exon 47 and c.5065T>C in exon 48) and a hemizygous *COL4A5* mutation (c.2767 + 11A>C in intron 32) were also detected. The mutations in the *COL4A4* gene were inherited from his father, and the mutation in the *COL4A5* gene was from his grandmother. The other two variants (c.5065T>C and c.2767 + 11A>C) were considered to be variants of uncertain significance (VUS). However, neither his father nor grandmother presented hematuria, proteinuria, or renal dysfunction and they had no extra-renal manifestations, such as sensorineural deafness and specific ocular lesions. Furthermore, the patient underwent audiometry, ophthalmological review, corneal examination and retinal optical coherence tomography. All results were normal. The diagnosis of LPG with CG is unquestionable.

**Fig. 3:**
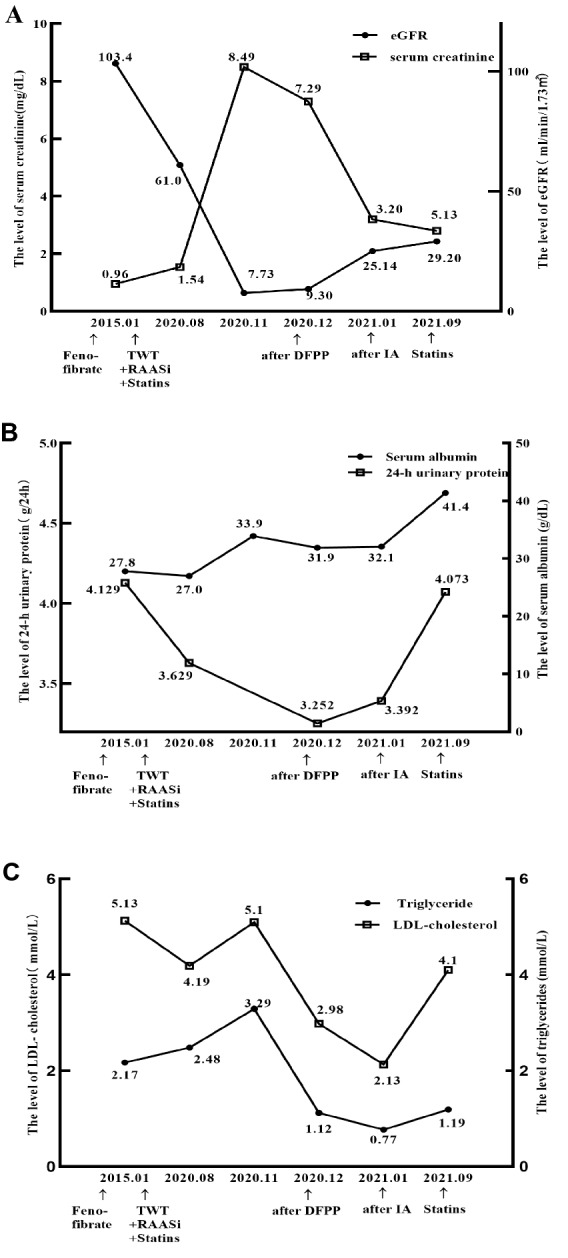
The dynamic changes in various parameters of this patient. **A** Changes in the levels of serum creatinine and eGFR (estimated by CKD-EPI formula). **B** Changes in the levels of 24-h proteinuria and serum albumin. **C** Changes in the levels of LDL-cholesterol and triglyceride. *TWT* *Tripterygium* *wilfordii* tablets; *RAASi* renin-angiotensin-aldosterone system inhibitors; *DFPP* double filtration plasmapheresis; *IA* immunoadsorption

The patient was treated with lipid-lowering agents, including fenofibrate and statins, renin–angiotensin–aldosterone system inhibitors and tripterygium glycosides (Fig. 3). However, he was hospitalized again in 2020 for deterioration of kidney function with elevated serum creatinine (8.49 mg/dL) and was diagnosed with AKI on CKD. There was no obvious predisposing factor inducing AKI, so the deterioration of renal function was considered to be caused by the primary disease. Double-filtration plasmapheresis (DFPP) was performed, and creatinine decreased mildly to 7.29 mg/dL after three rounds of therapy. DFPP was thus replaced by immunoadsorption (IA) and creatinine dropped to 3.20 mg/dL after eight rounds of IA and was stable at 2.81 mg/dL for a long period (Fig. 3A–C).

## Discussion

We report the first case of LPG and CG in the world. The patient was diagnosed with LPG and CG through laboratory examination, renal biopsy and WES results.

The patient presented with renal failure, elevated serum Apo E levels and the characteristic pathological manifestations of LPG. The glomerular capillaries were found to be distended by the lipoprotein thrombi under LM and EM. Immunofluorescence showed staining for APOA and APOB in the glomerular capillaries. Genetic testing further identified a pathogenic variant (c.127C>T; p. Arg43Cys) in exon 3 of the *APOE* gene inherited from his grandmother and mother without any clinical manifestations, which may be related to incomplete penetrance [7]. It has been defined as *APOE* -Kyoto of LPG [7].

To date, there is no specific treatment available for LPG. Glucocorticoids and immune suppressants were ineffective. DFPP and IA treatments were performed when the kidney function deteriorated. Immunoadsorption therapy seems more appropriate for this patient compared with DFPP. A search of the literature showed that IA and DFPP are the most common treatments for LPG, but which method is best is still controversial. Long-term follow up shows that IA can delay the progression of LPG. However, the decrease of immunoglobulin often leads to increase of infectious events. Therefore, since 2010, the National Clinical Research Center for Kidney Disease has suggested using DFPP to remove Apo E for the treatment of LPG, and DFPP has been identified as an effective method.

Renal pathology is the gold standard for the diagnosis of CG. LM and EM revealed that collagen III was deposited segmentally in the GBM and mesangial area. In addition, the serum PIIINP level, which is a noninvasive index for CG, was significantly increased [8]. CG may have a genetic basis, especially when presenting in childhood, but it is considered a sporadic disorder when it appears in adults [2]. No clear pathogenic gene was found. CG mainly occurs in elderly individuals, possibly because the accumulation of collagen occurs over a long time. Our case was diagnosed at the age of 21, which may be the result of interactions with the other kidney diseases.

There is no doubt about the diagnosis of LPG with CG in this patient. Although WES revealed three mutations in the *COL4A* gene, including two mutations in *COL4A4* on chromosome 2 inherited from his father, one of which was reported as a suspected pathogenic mutation and the other was a VUS. Another VUS was found in *COL4A5* on chromosome X inherited from his grandmother and mother. This genetic pattern does not conform to compound heterozygous inheritance or digenic disease that can cause Alport syndrome (ATS). In addition, the clinical and pathological manifestations of this patient do not support ATS, thus the diagnosis of ATS is not considered.

In conclusion, no confirmed cases of these two nephropathies occurring simultaneously have been reported in the literature. These rare diseases are related to metabolic abnormalities, and may highlight a close relationship between lipid metabolism and collagen synthesis.

## Data Availability

Not applicable.
